# Cell lineage branching as a strategy for proliferative control

**DOI:** 10.1186/s12915-015-0122-8

**Published:** 2015-02-19

**Authors:** Gentian Buzi, Arthur D Lander, Mustafa Khammash

**Affiliations:** Department of Biosystems Science and Engineering, Control Theory and Systems Biology, ETH Zurich, Basel, 4058 Switzerland; Department of Developmental and Cell Biology, University of California Irvine, Irvine, CA 92697 USA; Department of Biomedical Engineering, University of California Irvine, Irvine, CA 92697 USA; Center for Complex Biological Systems, University of California Irvine, Irvine, CA 92697 USA

**Keywords:** Cell fate, Control theory, Differentiation, Feedback control, Growth control, Homeostasis, Proliferative dynamics, Mathematical modeling, Robustness, Self-renewal, Stem cells, Tradeoffs

## Abstract

**Background:**

How tissue and organ sizes are specified is one of the great unsolved mysteries in biology. Experiments and mathematical modeling implicate feedback control of cell lineage progression, but a broad understanding of what lineage feedback accomplishes is lacking.

**Results:**

By exploring the possible effects of various biologically relevant disturbances on the dynamic and steady state behaviors of stem cell lineages, we find that the simplest and most frequently studied form of lineage feedback - which we term renewal control - suffers from several serious drawbacks. These reflect fundamental performance limits dictated by universal conservation-type laws, and are independent of parameter choice. Here we show that introducing lineage branches can circumvent all such limitations, permitting effective attenuation of a wide range of perturbations. The type of feedback that achieves such performance - which we term fate control - involves promotion of lineage branching at the expense of both renewal and (primary) differentiation. We discuss the evidence that feedback of just this type occurs *in vivo*, and plays a role in tissue growth control.

**Conclusions:**

Regulated lineage branching is an effective strategy for dealing with disturbances in stem cell systems. The existence of this strategy provides a dynamics-based justification for feedback control of cell fate *in vivo*.

See commentary article: http://dx.doi.org/10.1186/s12915-015-0123-7.

**Electronic supplementary material:**

The online version of this article (doi:10.1186/s12915-015-0122-8) contains supplementary material, which is available to authorized users.

## Background

The sizes of organs are often achieved with high precision, commonly being specified both genetically and autonomously (that is, size is controlled by tissues themselves, and not by their surroundings) [1,2]. Such observations imply that growing tissues ‘know’ the size they need to attain, but how this is implemented is not generally understood.

In engineering, set-point control is commonly achieved through feedback, especially ‘integral’ feedback, in which corrections to system behavior grow in magnitude the longer a system’s output differs from a set-point. The idea that feedback is important in tissue growth control is old [3], but only recently has it been shown that one particular form of feedback - in which the differentiated cells at the ends of lineages (‘terminal cells’) inhibit the self-renewal probability of the stem or progenitor cells that produce them - has the characteristics of an integral control circuit, and can thereby set a steady state or final tissue size that is insensitive to multiple types of perturbations [4].

The existence of this type of feedback has been documented in several cell lineages, including skeletal muscle, the olfactory epithelium, and the hematopoietic system [4-8]. In the first two cases, the direct mediators of feedback are secreted molecules of the tumor growth factor beta (TGF-β) superfamily. The fact that TGF-β family members suppress self-renewal (promoting differentiation) in other lineages as well (for example, [9-11]) suggests that this growth control strategy may be widespread, although the limited spatial ranges over which growth factors and other secreted signaling molecules act (typically 0.1 mm or less [12-17]) suggest that this type of local feedback most likely operates on the tissue, rather than the organ scale (for example, controlling epithelial thickness, or the sizes of compartments like intestinal crypts and epidermal appendages). Still, even if other feedback mechanisms come into play at larger scales (for example, mechanical forces, humoral factors), the general strategy of using a measure of differentiated cell content to feed back upon the renewal-versus-differentiation behavior of stem and progenitor cells - which we refer to here as renewal control - provides an appealing strategy for achieving set-point control of growth, as well as dynamics of growth and regeneration that fit with what is often observed *in vivo* [8,18,19].

**Figure 1:**
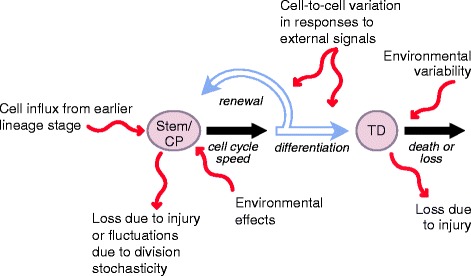
**Disturbances and their impact on the dynamics of a two-stage cell lineage.** Stem or committed progenitor (CP) cells can self-renew or differentiate to terminally differentiated (TD) cells. The processes of cell division, renewal or differentiation, and cell death can be affected by a number of biologically relevant disturbances that affect the steady state populations of terminal and stem or CP cells.

Despite the appeal of the renewal control strategy, there are reasons to expect that it comes at the expense of performance tradeoffs [20], that is, the cost of making tissue growth more robust to certain kinds of perturbations (for example, a subset of those illustrated in Figure 1) might be to make it more fragile to others. Here we show that this is indeed the case, regardless of whether continually renewing or fully differentiated tissues are being produced. In particular, we show that the high-gain feedback necessary for fast response and rejection of certain classes of disturbances invariably renders such systems less robust (or even unstable) in the face of other disturbances. Using tools from robust control theory, we show that the reasons for this limitation are structural, that is, they relate to the nature of the feedback strategy, not choices of parameters used to implement it.

Intriguingly, we find these tradeoffs can be alleviated through an alternate strategy that we refer to as fate control, whereby lineages branch - that is, stem or progenitor cells produce more than one type of differentiated product - and the branching decision becomes the target of feedback control. Remarkably, just such behavior was recently described in the olfactory epithelium, where two TGF-β family members, activin and GDF11, that mediate feedback control of neuron number were found to regulate the progression of neural stem cell progeny down a non-neuronal, supporting-cell lineage branch [5]. Indeed, lineage branching is a common feature of many tissues, both during development and regeneration [21-25]. We show here that such differentiation schemes solve an important, generic control problem in the feedback regulation of growth.

## Results

### Feedback regulation of stem cell renewal robustly stabilizes lineage pathways

**Figure 2:**
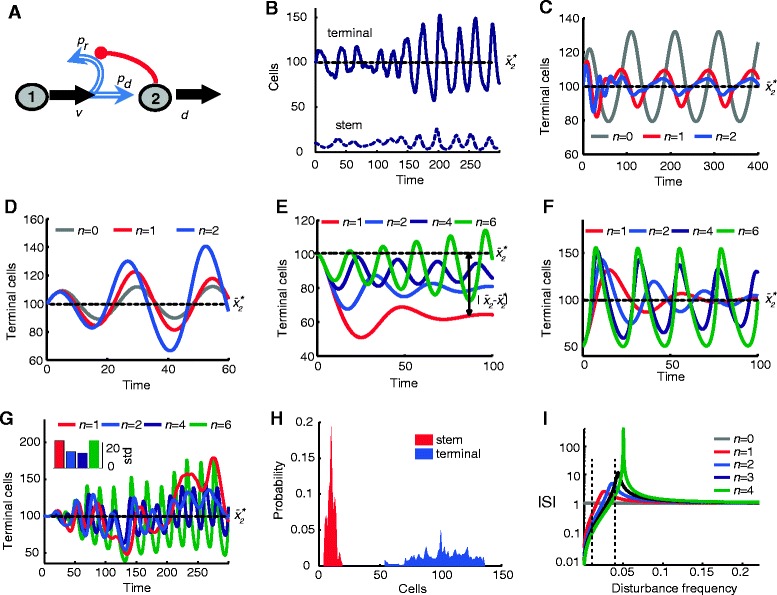
**Effects of disturbances on the performance of renewal control.** In all panels, time is given in units of cell cycles (normalized by *v*) and the system is at steady state at time *t* = 0. ${\overline{x}}_2^{*}$ is the desired (unperturbed) terminal cell population. For parameter values used see Additional file 1: Table S1. **(A)** Cartoon representation of renewal-controlled two-stage cell lineage. Red line represents negative feedback regulation of *p*_*r*_. **(B)** Shown is terminal cells (solid line) and stem cells (dashed line) response to stochastic fluctuations on *d* (*n* = 2). **(C**-**D)** Shown is stem cell population over time in response to periodic oscillations of *d* with frequency 0.01 (panel C) and 0.04 (panel D). Feedback suppresses the oscillations at low frequency (C) but amplifies them at higher frequency (D) **(E)** Plotted is the terminal cell population response to stem cell loss at a constant. Stronger feedback reduces steady state error but also introduces stronger oscillations. **(F)** Shown is the terminal cell population after abrupt removal of half the terminal cell population *x*_*2*_ at *t* = 0. Aggressive feedback has faster rise time but causes oscillations in terminal cell population. **(G)** Main plot shows terminal cell response to stochastic disturbances directly affecting the stem cell population. The inset shows the standard deviation (std) of responses for each feedback level. Moderate feedback can reduce the variance, but more aggressive levels increase variance. **(H)** Shown is the distribution of stem and terminal cell populations for the response shown in (G) with *n* = 2. **(I)** Shown is the plot of the sensitivity function *S* for renewal control as a function of disturbance frequency (normalized by *v*). Aggressive feedback improves performance at low frequency (smaller |*S*|) but by necessity this results in poorer performance at a higher frequency range (larger |*S*|). For additional disturbances see Additional file 1: Figure S2.

We begin by considering the simplest example of renewal control, in which feedback acts upon a stem cell (type 1) whose progeny either remain stem cells or differentiate into terminal, post-mitotic (type 2) cells (Figure 2A). We let *v* stand for the rate of cell division (that is, the cell doubling time is ln 2/*v*); *p*_*d*_ for the probability, at each division, that daughter cells differentiate; *p*_*r*_ for the probability, at each division, that daughter cells remain stem cells (hence *p*_*r*_ =1- *p*_*d*_); and *d* for the probability, per unit time, that terminal cells die. If we let *x*_*1*_ and *x*_*2*_ stand for the concentrations (or numbers) of stem and terminal cells, respectively, then for large enough cell numbers, the dynamics of the system may be described by a pair of ordinary differential equations:

1 $$ \begin{array}{l}{\dot{x}}_1=\left(2{p}_r\left({x}_2\right)-1\right)v{x}_1\\ {}{\dot{x}}_2=2{p}_d\left({x}_2\right)v{x}_1-d{x}_2\end{array} $$

in which renewal control is represented by the fact that *p*_*r*_ and *p*_*d*_ are taken to be functions of *x*_2_ (that is, levels of differentiated cells influence the renewal or differentiation choice of stem cells), subject to the condition *p*_*r*_(*x*_*2*_)* + p*_*d*_(*x*_*2*_)* =* 1*.* The form of each equation derives from the fact that the rate of production of each cell type occurs at the rate of the stem cell cycle multiplied by two (because two daughter cells are produced with each division) times the probability that a stem cell daughter becomes either a stem cell (*p*_*r*_) or a differentiated cell (*p*_*d*_). The additional ‘-1’ in the first equation reflects the fact that, with each stem cell division, one ‘mother’ cell disappears. This model reduces to the one used by [4], if the parameter *p*_*r*_ is replaced *p* and *p*_*d*_ by 1-*p.*

For constant values of *p*_*r*_ and *p*_*d*_, system 1 trajectories necessarily blow up for *p*_*r*_ > 0.5 and converge to zero for *p*_*r*_ < 0.5. A stable steady state requires negative feedback, that is, *p*_r_(*x*_*2*_) must be a declining function of *x*_*2*_ around the value of *x*_*2*_ at which *p*_*r*_(*x*_*2*_) = 0.5. Such feedback produces not only stability, but also robustness, in the sense that the level of terminal cells in the system at steady state becomes determined only by the relationship between *x*_*2*_ and *p*_*r*_, and not by the other parameters of the system, *v* and *d*, or by initial conditions. Such ‘perfect’ robustness is a result of integral-like control implemented by the feedback loop, with integrator *σ* = ln*x*_1_ and error *e* = (2*p*_*r*_(*x*_*2*_)-1)*v* (as alluded to in [4].)

For some steady state properties of system 1, the exact shape of the feedback function is irrelevant, but to understand dynamic behaviors or responses to external perturbations, the details are important, particularly the steepness, or ‘aggressiveness’ with which *p*_*r*_ changes with *x*_2_. If we let *k*_r_ stand for the propensity of stem cells to self-renew, and *k*_d_ their propensity to differentiate, then the net renewal probability *p*_*r*_ can be written as *k*_*r*_/(*k*_*r*_ + *k*_*d*_) = 1/(1 + *k*_*d*_/*k*_*r*_). In other words, *p*_*r*_ can be thought of as a monotonic function of the ratio between underlying propensities to differentiate and renew. In the simulations presented here, we take this ratio to be $0.5+0.5{\left({x}_2/{\overline{x}}_2\right)}^n$, such that parameter ${\overline{x}}_2$ corresponds to the value of *x*_2_ at which stability (balanced renewal and differentiation) is achieved, and parameter *n* captures the feedback gain (steepness). This parameterization approximates most smooth, monotonic saturating functions well enough that the qualitative behaviors we report below hold true even when *p*_*r*_ is defined in a variety of other ways. This is because, as we show, the critical feature for these behaviors is the slope of *p*_*r*_ near the steady state, which is directly related to the aggressiveness (gain) of the feedback.

### Renewal control is sensitive to many types of biologically relevant disturbance

As shown in Figure 1, stem cell systems potentially face many kinds of perturbation. ‘Active’ stem cells may be recruited from ‘quiescent’ populations, and stem cells may undergo cell death, or become quiescent themselves. The speed of the cell cycle may vary from cell to cell, and may be influenced by environmental factors (for example, proximity to nutrients, temperature gradients). Environmental inputs may also affect the decision to renew or differentiate, but even without environmental forcing, stochastic effects that result from symmetric patterns of divisions (where both stem cell progeny adopt the same fate, as occurs commonly in many mammalian tissues) will create substantial fluctuations in stem cell pool sizes [26]. Rates of loss of terminally differentiated stem cell progeny can also be expected to fluctuate because of disease, injury, or variable patterns of organ use. For example, in the olfactory epithelium, in which feedback controls neuron number [4,5,27], the rate of neuron turnover is highly dependent on the environment in which animals are reared [28].

Figure 2 illustrates the effects of some of these kinds of perturbations on the behavior of a renewal-controlled lineage (system 1, as shown schematically in panel A). For example, simulations in panels B-E explore the effects of a time-varying rate of loss of terminal cells (parameter *d*). In panel B, stochastic noise is added to this loss rate (here the gain of the feedback is taken to be *n* = 2). Notice how, even though the system’s steady state behavior would be perfectly robust to a constant step change in *d*, it does not compensate well for fluctuating *d*. In panels C and D, the fluctuations imposed on *d* are oscillatory, at fixed frequencies, and the effect of different feedback gains is considered (in engineering it is common to characterize the responses of systems to sinusoidal perturbations because, as discussed below, time-varying perturbations can always be decomposed into sums of sinusoids). The results show that increasing feedback gain helps suppress the effects of low frequency oscillations in *d*, but amplifies the effects of high frequency ones.

It is not necessary to use fluctuating or periodic disturbances to bring out the limitations of high-gain feedback. For example, in Figure 2E, the system was subjected to a constant loss of 10% of stem cells per cell cycle (for example, representing stem cell death, or diversion of stem cells to differentiate in an alternate way). This produces a steady state error in the number of terminal cells. With increasing feedback gain the error can be diminished (it is straightforward to show that the steady state error - that is, the difference between the unperturbed and perturbed steady state populations of stem cells, see Box 1 for details - is equal to $\left(1-{0.636}^{1/n}\right){\overline{x}}_2$), but this occurs only at the expense of producing ever larger oscillations in terminal cell numbers.

Figure 2F models the response to an acute injury that removes a large proportion (half) of all terminal cells. With increasing feedback gain, the system can recover its terminal cells (that is, regenerate) ever more rapidly, but only at the expense of larger, longer-lasting oscillations. Indeed, for high enough gain (*n* > 4), oscillations become so large that, effectively, no steady state is achieved. In tissues that rapidly regenerate after just such injuries, for example, the olfactory epithelium and the liver, such large swings in terminal cell number are not observed [18,19] although, interestingly, marked oscillations in terminal cell numbers do happen in some tissues [29], as well as in a variety of pathological conditions [30-33].

Finally, Figure 2G,H models the impact of stochasticity in stem cell numbers, as might be expected to arise due to random patterns of division. Such fluctuations can be large for small, compartmentalized stem cell pools (for example, intestinal crypts, in which random symmetric divisions drive frequent extinction of stem cell clones [34]) but may even be significant in large, non-compartmentalized epithelia. This is because secreted factors that mediate lineage feedback will have limits to their spatial range, so that if most cells tend to remain close to where they were generated, feedback will be effectively compartmentalized, even if there are no fixed boundaries. In these simulations, stochasticity was introduced through adding a noise term (birth-death process) to the right hand side of the first equation of system 1. As the results show, for low feedback gains, feedback reduces the variation in stem and terminal cell numbers, but with increasing gain this effect reverses, and the variance increases. At the highest gains (when feedback is effectively switch-like), the probability distribution for terminal cell numbers becomes very broad (Figure 2H).

Simulations of the effects of other types of disturbances show similar tradeoffs between the ability of high feedback gains to reject disturbances and the ability of the system to avoid oscillation and instability (Additional file 1: Figure S2). These include constant increases to stem cell numbers (as might reflect the recruitment of quiescent cells) or fluctuations in the parameters underlying the feedback function itself (for example, the intrinsic propensity of cells to differentiate or renew), as might reflect normal variability in the levels of cellular proteins that drive cellular decision-making.

### Performance limitations are imposed by the structure of the lineage pathway

In the above examples, steady state errors created by some, but not all, disturbances to a renewal-controlled lineage can be reduced by aggressive feedback, but high feedback gains also bring on oscillations and instability. What accounts for such behavior? Is it a result of particular choices of parameters, or the means of realizing the feedback?

The tools of robust control theory provide a way to address such questions. Briefly, for a given system, one can calculate a frequency-dependent function *S* - the sensitivity function - that captures the ability of feedback control to attenuate disturbances of different frequencies (see Box 1). When |*S*| < 1, feedback diminishes the effect of a perturbation; when |*S*| > 1 it amplifies it. Because time-varying disturbances can be decomposed into components of different frequencies, a plot of |*S*| versus frequency provides a global view of performance. Such a plot is shown for system 1 in Figure 2I, for different values of feedback gain. In all cases, feedback attenuates low-frequency (slowly changing) disturbances, but amplifies high-frequency (rapidly changing) ones. The higher the gain, the better the performance at low frequencies and the worse the performance at high ones. This suggests that ‘hard limits’ underlie the behaviors observed in Figure 2B-H. Indeed, as shown in Box 2 (and explained further in Additional file 1: Supporting Information), the architecture of system 1 ensures that |*S*| must obey a conservation law, implying that |*S*| cannot be made small for all frequencies simultaneously. In effect, improved performance (smaller |*S*|) for a certain range of frequencies necessarily implies poorer performance (larger |*S*|) for some other range of frequencies. The existence of this tradeoff is independent of both the parameters of the system and the way feedback is realized, that is, the functional form of *p*_r_(*x*_2_).

A second limitation imposed solely by the structure of the system involves a relationship between the aggressiveness of feedback and stability (the ability to reach a steady state). As shown in Figure 2I, beyond a certain level of gain, perturbation frequencies exist at which |*S*| approaches infinity (a phenomenon known as high-gain instability). This corresponds to the observation, seen in Figure 2E-G, of sustained oscillations when feedback gains are sufficiently high (for example, *n* > 4).

In Figure 2I, disturbance frequencies are expressed in time units equal to the length of the stem cell cycle. Thus, one way to make the system better able to reject higher-frequency perturbations would be to speed up cell division so that, relative to the cell cycle, any given perturbation would be of lower frequency. Indeed, introducing additional negative feedback control of cell cycle speed *v* - as does occur with GDF11 in the olfactory epithelium [4] - can modestly extend the range of disturbance frequencies over which robust control can be achieved. It does not, however, remove the performance limitations imposed by the system’s structure (see Additional file 1: Supporting Information for further details). Such limitations also persist if the length of the lineage is increased (by adding an intermediate cell stage to the pathway).

### Removing performance limitations through controlled pathway branching

The reason the performance limitations discussed above are inherent in the structure of system 1 is that the probabilities of renewal and differentiation are necessarily coupled. With stem cells able to make only two choices at each division, negative regulation of one of them implies positive regulation of the other. But stem and progenitor cells could have other choices. They could differentiate along an alternate trajectory, producing a different cell type, becoming quiescent, or even choosing to die. Such choices create branches in cell lineages, and examples of such branching exist in both vertebrates and invertebrates, during development and regeneration, and in both solid and blood-borne tissues [21-25]. Even in tissues in which lineage branching has not been demonstrated directly, the observation that different cell types appear in temporal waves (either during development or regeneration) is consistent with the model of a common stem or progenitor cell that gradually alters its differentiation choices over time (for example, [35,36]).

**Figure 3:**
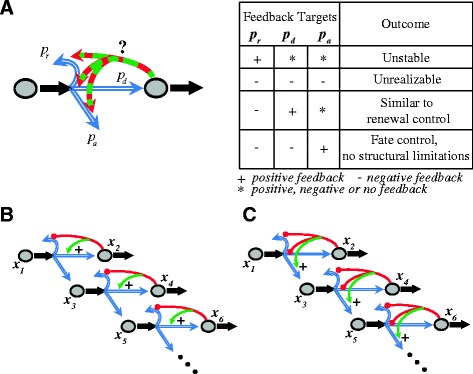
**Control strategies and layered architectures. (A)** Lineage branching allows for different control strategies, depending on how the probabilities of differentiation and replication are regulated. Any strategy with a positive regulation of *p*_*r*_ is unstable. Negative feedback on all the probabilities is unrealizable because the probabilities must add up to 1. Any strategy implementing negative feedback on *p*_*r*_ and positive feedback on *p*_*d*_ is akin to renewal control. The last strategy, which implements positive feedback on *p*_*a*_ with simultaneous negative feedback on *p*_*r*_ and *p*_*d*_, is free from the limitations described in the text. We refer to this feedback strategy as fate control. **(B)** Multi-layer cell lineage with renewal control strategy. **(C)** Multi-layer cell lineage with a fate control strategy. Red lines represent negative feedback regulation, and green arrows represent positive feedback regulation.

The behavior of the simplest system in which a stem cell under feedback control by descendants of one cell type (‘type 2’) can choose stochastically to switch to production of an alternate differentiated cell type (‘type 3’) is:

2 $$ \begin{array}{l}{\dot{x}}_1=\left(2{p}_r\left({x}_2\right)-1\right)v{x}_1\\ {}{\dot{x}}_2=2{p}_d\left({x}_2\right)v{x}_1-d{x}_2\\ {}{\dot{x}}_3=2{p}_a\left({x}_2\right)v{x}_1-{d}_3{x}_3\end{array} $$

in which *p*_a_(*x*_2_) stands for the probability of choosing the type 3 fate. Because type 3 cells are not taken to have any influence on the production of type 1 or type 2 cells, the first two equations are identical to those in system 1; indeed if the alternative cell fate is death, then the third equation can be omitted altogether. What is significantly different about system 2 is that the condition *p*_*r*_(*x*_2_) + *p*_*d*_(*x*_2_) = 1 no longer holds, being replaced by *p*_*r*_(*x*_2_) + *p*_*d*_(*x*_2_) + *p*_*a*_(*x*_2_) = 1. This means that regulation of *p*_r_(*x*_2_) in one direction no longer guarantees that *p*_d_(*x*_2_) must change in the opposite direction. The possible ways in which *p*_r_(*x*_2_) and *p*_d_(*x*_2_) may now be jointly regulated are enumerated in Figure 3A.

**Figure 4:**
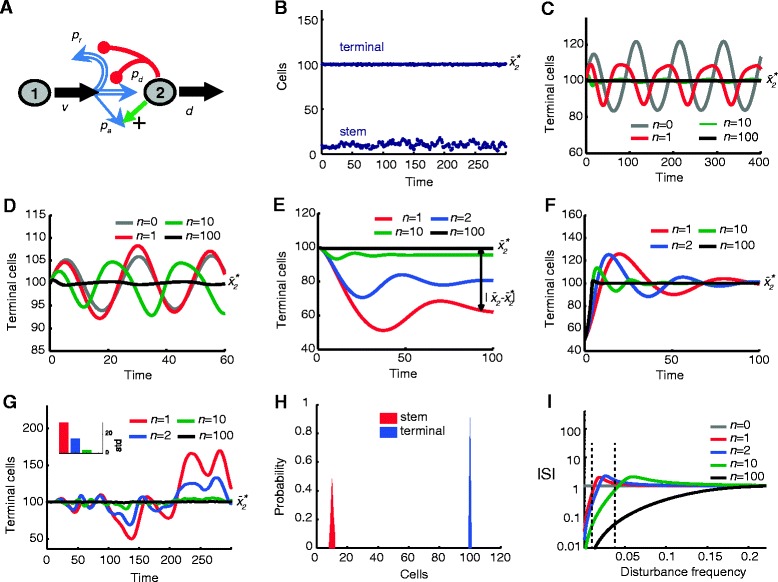
**Effects of disturbances on the performance of fate control.** In all panels, the system is at steady state at time *t* = 0, and larger *n* corresponds to stronger feedback. For parameter values used see Additional file 1: Table S1. **(A)** Cartoon representation of fate-controlled two-stage lineage. Stem cell progeny can remain stem cells, differentiate into terminal (type 2) cells, or differentiate into alternate terminal cells. *p*_*a*_ is the probability of differentiation to alternate terminal cells (*p*_*a*_ = 1 - *p*_*r*_ - *p*_*d*_). Red lines represent negative feedback regulation on *p*_*r*_ and *p*_*d*_, and the green arrow represents positive feedback on *p*_*a*_. **(B)** Shown is the population of terminal cells (solid line) and stem cells (dashed line) in response to stochastic fluctuations on *d* (*n* = 2). **(C**-**D)** Shown is stem cell population response to periodic oscillations of *d* with frequency 0.01 (panel C) and 0.04 (panel D). Feedback suppresses oscillations due to both disturbance frequencies. **(E)** Plotted is terminal cell population response to stem cell loss at constant rate*.* Stronger feedback reduces steady state error without causing oscillations. **(F)** Shown is the terminal cell population after abrupt removal of half the terminal cell population at *t* = 0. More aggressive feedback has faster rise time and less transient oscillations. **(G)** Main plot shows terminal cell populations response to a stochastic disturbance directly affecting the stem cell population. The inset shows the standard deviation (std) of responses for each feedback level. Aggressive feedback reduces the variance of the response. **(H)** Shown is the distribution of the stem and terminal cell populations for the response shown in (G) with *n =* 100. **(I)** Shown is the plot of the sensitivity function *S* for fate control as a function of disturbance frequency. Aggressive feedback improves performance (smaller |*S*|) across all frequencies. For additional types of disturbances see Additional file 1: Figure S3.

The first two strategies shown are either unstable or unrealizable. The third strategy is, essentially, renewal control, and suffers from all the limitations discussed above. The last strategy, however, in which there is positive feedback regulation of *p*_a_ with simultaneous negative regulation of *p*_r_ and *p*_d_, behaves dramatically differently. A cartoon representation of system 2 with this feedback strategy - which we refer to here as fate control - is shown in Figure 4A.

As before, we motivate our choice for the form of the feedback function by considering that the three probabilities, *p*_r_, *p*_d_, and *p*_a_, arise from independent propensities, *k*_r_, *k*_d_, and *k*_a_, to self renew, differentiate into type 2 cells, or differentiate into type 3 cells, respectively. With positive feedback on *k*_a_, that is, *k*_a_(*x*_2_) that is monotonically increasing in *x*_2_, there can now be negative feedback on both *p*_r_ and *p*_d_, because:

3 $$ {p}_r=\frac{k_r}{k_r+{k}_d+{k}_a\left({x}_2\right)},\kern1em {p}_d=\frac{k_d}{k_r+{k}_d+{k}_a\left({x}_2\right)} $$

For simplicity, we consider here the situation in which *k*_r_ and *k*_d_ are not themselves regulated, so that they maintain a fixed ratio *κ* = *k*_d_/*k*_r_; consequently *p*_d_ = *κp*_r_. In most numerical simulations we take ${k}_r=1,{k}_d=0.5,\kern0.5em {k}_a\left({x}_2\right)=0.5{\left({x}_2/{\overline{x}}_2\right)}^n$. However, as before, the results are not particularly sensitive to this parameterization. This is because, as before, the critical feature is the slope of *p*_r_ and *p*_d_ near the steady state, which is directly related to the aggressiveness of the feedback.

Figure 4 illustrates the performance of this system in exactly the same disturbance scenarios that were explored for renewal control in Figure 2. The previously identified performance tradeoffs and instabilities are now gone. Fluctuations in the turnover rate of type 2 cells are effectively suppressed by high-gain feedback without introducing any oscillatory tendency (Figure 4B-D). High feedback gain also enables the system to compensate for both a constant rate of loss of stem cells, and an abrupt removal of terminal cells, without the risk of instability in either case (Figure 4E,F). Such feedback also prevents stochastic fluctuations in stem cell numbers from propagating to terminal cell numbers (Figure 4G,H).

The reason for these dramatic improvements is evident in the plot of |*S*| versus disturbance frequency (Figure 4I). No longer does a conservation law require reductions in |*S*| at some frequencies to be balanced by increases in |*S*| at others. No longer does high feedback gain promote instability. As a result, high gains can be used to enable responses to disturbances that are arbitrarily fast (gains up to *n* = 100 are considered in Figure 4C-H). Under such conditions, the response to a deficit of terminal cells of type 2 would be for stem cells to renew and generate cells of type 2 at a constant, high rate until a sufficient number were produced, then rapidly switch to generating cells of type 3.

### Branched lineages and layered architectures

Branched lineages can escape the performance limitations of unbranched ones because, through fate control, they can realize negative feedback on both the probability of replication (*p*_*r*_) and the probability of differentiation into terminal cells (*p*_*d*_). But what about the alternative fate that the lineage branch creates? In the event that that fate is not cell death, will the numbers of such cells be well controlled? In system 2, with *p*_r_ a decreasing function of *x*_2_, we see that the value of the alternate cell population (*x*_*3*_) always reaches a steady state, but unlike *x*_*2*_, it is not robust, being linearly dependent on *d*/*d*_*3*_, the ratio of the turnover rates of *x*_*2*_ and *x*_*3*_. Moreover, cell type 3 is highly ‘exposed’ to disturbances, as disturbances on the other cell types directly affect the dynamics of *x*_3_, and there is no direct influence of *x*_3_ on its own value. Put another way, whereas the existence of cell type 3 can help solve some of the control problems faced by cell type 2, controlling cell type 3 requires additional mechanisms (and those mechanisms should not interfere with the control of cell type 2).

If cell type 3 is a terminally differentiated cell, there are not too many options, other than to regulate its turnover. But if it is a progenitor cell, it could receive negative feedback from its own differentiated progeny, much like cell type 1 does. We refer to this as a ‘layered’ lineage architecture because one layer (progenitor cell type 1 fed back upon by differentiated cell type 2) branches to give rise to a second layer (progenitor cell type 3 fed back upon by differentiated cell type 4). Of course, we might expect that negative feedback at the second layer will be subjected to the same challenges and tradeoffs discussed for simple unbranched lineages (that is, system 1), unless that layer itself branches, creating a progenitor cell type 5 that is fed back upon by a differentiated cell type 6.

Indeed, one could imagine a succession of many layers, each controlling itself through feedback, and branching to produce the next layer. In fact, there are many *in vivo* systems in which the dynamics of cell type production and progenitor cell potency suggest the existence of such layered architectures (see Discussion). To explore their control implications, we compare two different layered scenarios: in one (Figure 3B), negative feedback occurs at each stage only through down-regulation of *p*_*r*_ and up-regulation of *p*_*d*_ (that is, renewal control). In the other (Figure 3C), feedback at each stage occurs through up-regulation of *p*_*a*_, with concomitant down-regulation of both *p*_*r*_ and *p*_*d*_ (fate control).

Analysis of steady state behavior indicates that either strategy improves parametric robustness (insensitivity to parameters), although perfect robustness exists only at the first layer. At each subsequent layer, integral feedback control is ‘leaky’, due to the (uncontrolled) influx of cells from the previous layer. The size of this leak at any given level can be kept small by having the influx from the previous level be small compared with the expansion of progenitors at that level.

**Figure 5:**
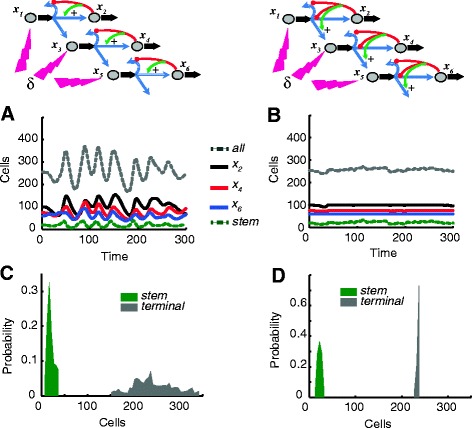
**Control of multi-layered lineages. (A, B)** Shown are populations of the different cells types over time in response to a stochastic forcing function in all three types of stem-like cells *x*_*1*_ *, x*_*3*_ *,* and *x*_*5*_ for a renewal-controlled multi-layer lineage (panel A) and a fate-controlled multi-layer lineage (panel B). **(C)** Histogram of the distribution of the total population of terminal and stem cells for the sample path in panel A. **(D)** Histogram of the distribution of the total population of terminal and stem cells for the sample path in panel B. For parameter values used see Additional file 1: Table S2.

In terms of responses to the sorts of disturbances described in Figures 2 and 4, the renewal control and fate control strategies perform very differently. Figure 5 illustrates responses to the same kind of stochastic disturbances that were explored in Figures 2B and 4B, applied now to all three stem/progenitor cell types in a three-layer branched lineage. With renewal control (Figure 5A,C), disturbances are transmitted to and may even be amplified at the level of the terminal cells. By contrast, with fate control (Figure 5B,D), the effects of such disturbances can be damped out (for these simulations, a feedback gain of *n* = 2 was used in the renewal control example, as it gave the best control, whereas a gain of *n* = 100 was used in the fate control example). These results closely mimic what was seen in Figures 2B and 4B, and the reasons are the same: fate control can exploit high-gain feedback to suppress disturbances, whereas renewal control cannot.

### Branched lineages and ‘final state’ tissues

Not all tissues arrive at a final size through a balance between cell production and cell loss. Many tissues are generated by stem cells that exist only transiently during development, producing terminal cells that survive for the life of the organism. For example, most of the mammalian nervous system falls into this category [35]. Such self-terminating, or ‘final state’ tissues can be modeled using the same approach as for steady state tissues, by simply setting the turnover rates for terminal cells to zero [4]. For a simple, unbranched, one-stage lineage with renewal control*,* nearly perfect robustness of final terminal cell numbers can be observed [4], demonstrating that renewal control provides integral feedback in final state tissues as well as steady state ones.

Should the effects of perturbations on models of final state tissues also resemble what occurs in steady state models? Intuition suggests that terminal cell oscillations will not occur in final state models, because the oscillations in steady state models depend upon cell turnover (that is, they are ‘relaxation’ oscillations). Instead, the consequence of overshooting a goal should simply be an irremediable overproduction of terminal cells.

**Figure 6:**
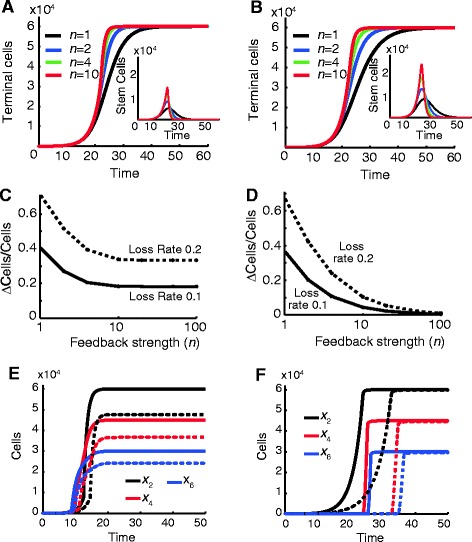
**Control of final state systems. (A, B)** Shown are populations of terminal cells over time for renewal control (A) and fate control (B) for a two-stage lineage. The inset shows the corresponding stem cell populations. Large feedback gains (n) do not significantly alter development time. The initial population consists of 10 stem cells of type *x*_*1*_ and no other cell types. **(C, D)** Shown is the error (deviation from desired final concentration divided by the desired final concentration) in development as a function of *n* in response to constant loss rates *ρ* = 0.1 and *ρ* = 0.2 of stem cells for the renewal control (C) and fate control (D). For renewal control, the benefits of feedback are modest. For fate control, sufficiently high gain can suppress the effects of stem cell loss nearly completely. **(E, F)** Shown are populations of the different terminal cell types *x*_*2*_, *x*_*4*_, *x*_*6*_ in the multi-layered topology. Solid lines show population trajectories when there is no disturbance, and the dashed lines the trajectories when there is loss of all stem-like cells *x*_*1*_, *x*_*3*_, *x*_*5*_ at rate *ρ* = 0.1. For renewal control (E), stem cell loss does not affect development time, but it results in smaller concentration of terminal cells (smaller tissue/organ) at every feedback level. For fate control (F), stem cell loss causes a delay in development time, but for high feedback gain the concentration of terminal cells is preserved. For panels A and C, *p*_*r*_ *=* 1/(0.5 + 0.5(*x*_*2*_/*b*_*n*_)^*n*^), *b*_*n*_ = 23859, 25726, 27816, 29505, 29867, 29971, and *29985* for *n* = 1, 2, 4, 10, 20, 50, and 100 respectively. For panels B and D, *p*_*r*_ = 1/(0.5 + 0.5(*x*_*2*_/*b*_*n*_)^*n*^), *p*_*d*_ = 0.5*p*_*r*_, *b*_*n*_ = 29990, 34636, 40121, 47206, 51526, 55462, and 57294 for *n* = 1, 2, 4, 10, 20, 50, and 100 respectively. For parameter values used in panels E and F see Additional file 1: Table S2.

To explore, quantitatively, the effect of feedback and branching on final state systems, we studied the same three-layer lineages and feedback strategies shown in Figure 5, but set cell turnover rates to zero. Figure 6A,B shows the dynamics of production of the initial terminal cell type (type 2 cells), either using renewal control or fate control at different feedback gains. Parameters were chosen to produce the same final numbers in both cases. We note that the total number of cell divisions does not differ substantially between the two cases.

In Figure 6C,D we explore the effects of a constant perturbation to stem cells (type 1 cells), by imposing a steady loss rate on *x*_*1*_, similar to what was done in Figures 2E and 4E. As anticipated, oscillations are not seen, but final state errors in the numbers of terminal (type 2) cells occur. These are plotted in Figure 6C,D as a function of the feedback gain. Here we see that high gain improves performance for both control strategies, but with renewal control (C) the effect is modest at best, with the final disturbance to terminal cell numbers actually exceeding the fractional rate of stem cell loss. By contrast, with fate control (D), a sufficiently high gain can suppress the effects of stem cell loss nearly completely (up to reasonable loss rates).

Figure 6E,F shows the dynamics of terminal cell production for a three-layered topology when there is no disturbance present and when there is loss of stem-like cells at each of the layers. One noticeable difference is that fate control (F) generates terminal cell types in distinct temporal waves, whereas with renewal control (E), all cell types appear more or less at the same time. Furthermore, with renewal control the timing of cell type production does not change substantially when there is stem cell loss, whereas with fate control the production of terminal cell types is markedly delayed (even though this strategy eventually does a much better job of producing desired final cell numbers).

## Discussion

Feedback regulation of cell lineage progression has recently been recognized as an important strategy for the control of tissue growth and homeostasis (for example, [4,6,8,37]). Negative regulation of stem or progenitor cell renewal by differentiated progeny - a strategy we refer to here as renewal control - can stabilize stem cell numbers, speed regeneration, and establish tissue sizes that are robust both to growth parameters (cell cycle speed, cell turnover) and initial conditions [4,38]. Yet, as we demonstrate here, this strategy also limits the ability of tissues to deal with a variety of realistic perturbations, including losses or additions to stem cell pools, or time-varying disturbances to cell numbers or growth parameters. By contrast, feedback regulation of lineage branching can circumvent these limitations. We refer to fate control as the strategy by which differentiated cells drive stem or progenitor cells to differentiate along a secondary fate pathway, at the expense (at least in part) of stem or progenitor cell renewal. The latter stipulation ensures that fate control also incorporates negative feedback on renewal, which is why it still achieves the desirable stability and robustness properties of renewal control, even while escaping the latter’s limitations.

Fundamentally, the limitations of renewal control arise because of the unavoidable coupling between renewal and differentiation, which causes responses to perturbations initially to go in a ‘wrong’ (that is, anti-homeostatic) direction. For example, with renewal control, an excess of differentiated cells will eventually restore their numbers through suppression of stem cell renewal (which lowers the number of stem cells). But because the only alternative to renewal is differentiation, reducing renewal will always initially lead to an increase in differentiated cell numbers, the opposite of a restoring effect. Such maladaptive dynamics are characteristic of what, in control theory terminology, is referred to as a ‘non-minimum phase’ system, and it is straightforward to show that an unbranched, renewal-controlled lineage is structurally non-minimum phase. By contrast, a fate control strategy is minimum phase, because differentiation (down a primary fate pathway) and renewal can be suppressed simultaneously (through up-regulation of progression toward an alternative fate). Control engineers make considerable efforts to avoid non-minimum phase systems, as they are generally subject to undesirable tradeoffs, the most serious of which can be captured by conservation laws requiring that the attenuation of some disturbances must come at the expense of the amplification of others. The presence of such tradeoffs for renewal control is clearly shown in Figures 2, 5, and 6. One of the most severe tradeoffs is that increasing feedback gains, which might otherwise help with the rejection of disturbances, can lead to increasing oscillation and, ultimately, instability. By contrast, with fate control, high gains (for example, switch-like responses) are never problematic (and often desirable).

In view of these findings, one might expect fate control to be especially prevalent in biology. Identifying it in biological data requires showing both that a lineage is branched, and that feedback promotes differentiation toward an alternative fate. Many lineages are indeed branched, including ones in the hematopoietic system, the nervous system, and connective tissue [22-25]. Even in tissues in which lineage relationships are not fully understood, the sequential appearance of cell types over time, during development or regeneration, often suggests lineage branching (for example, [39]). Moreover, hematopoietic and neural lineages are commonly depicted in terms of layered branching architectures similar to those in Figure 3 [21,23]. It should be noted that nothing about fate control as a feedback strategy requires layered lineages to be as deterministic and irreversible as suggested by Figure 3: even an architecture in which a multipotent stem cell always has some probability of producing every differentiated cell type, and simply gradually changes those probabilities as different cell types are produced, should behave similarly - from the standpoint of control - to an architecture with fixed layers.

Evidence that feedback factors produced by terminal cells promote fate transitions is harder to come by, but observations in at least two systems - the olfactory epithelium and the neural retina - support this view. In the mouse olfactory epithelium, it was recently shown that a single type of stem cell acts as the precursor of both the committed progenitors of neurons and sustentacular (glial) cells [5]. Activin βB, which is produced by the neuronal cells of the epithelium, feeds back onto this stem cell to inhibit neuronal, and favor glial, differentiation. The fact that stem cell numbers rise in activin βB-null mutant mice implies that activin not only promotes glial fate choice but also decreases stem cell renewal, as required by an effective fate control strategy. The molecular mechanism of activin action in this tissue provides a likely explanation for its fate-controlling effect, as it targets for rapid degradation the transcription factor Ascl1, which is known to drive neuronal fate choice (in this tissue and in others [5,40-42]).

In the mouse retina, ganglion cells (one of the earliest-generated neuronal cell types) produce GDF11, which feeds back on multipotent retinal progenitors to decrease the probability that their offspring adopt a ganglion cell fate, and increase the probability that amacrine and photoreceptor cells (which normally appear after ganglion cells) are produced [43]. The production of still other cell types of the neural retina in an orderly sequence suggests that other fate-controlling signals either direct sequential lineage branching (for example, as diagrammed in Figure 3), or gradually alter the alternate fates available at the end of a single lineage branch (as recently proposed [44]). The molecular nature of such signals has yet to be discovered, however.

Studies in the intestine also provide evidence for fate control as a strategy for regulating cell numbers. In mouse intestine, the effects of genetic ablation of bone morphogenetic protein (BMP) receptors from the intestinal epithelial lineage indicate that the BMPs produced by one class of terminal cells (enterocytes, or absorptive cells) feed back to promote differentiation of a common progenitor into enteroendocrine cells, an alternate lineage branch [45]. That this feedback is involved in the control of terminal cell numbers is demonstrated by the increased number of dividing cells per crypt, and increased villus length, in the receptor-mutant animals.

Other examples of cell number control via fate control are likely to exist, especially if one expands the notion of fate choice to include cell emigration or death. For example, in the hematopoietic system, regulated stem cell death is known to play an important role in controlling steady state cell numbers [46], and it has been suggested that mobilization of stem cells from the marrow to the blood serves a similar homeostatic role [47]. The high rate of progenitor cell death that is observed in lymphoid lineages [48] could also play a role in numerical homeostasis, outside of its known role in selection for appropriate antigen-reactivity. Even in the nervous system, it has recently been argued that regulated progenitor cell death plays an important role in tissue size control [49].

As discussed above, one of the advantages of a fate control feedback strategy is that it allows for the use of high gain (that is, steep feedback) to suppress disturbances. Although it is not simple to measure gains *in vivo*, recent observations in the developing mouse intestinal epithelium [36] support the view that, in at least some cases, gains are indeed high: in that tissue, postnatal development is characterized by a period of nearly pure exponential expansion of stem cells, which switches abruptly to one of stem cell homeostasis (stabilizing enterocyte production at a roughly constant rate). Switching occurs in each crypt as it reaches a threshold size, suggesting control by local feedback. The abruptness of the switch (the extent to which stem cell renewal does not decrease until crypts have reached nearly their full complement of stem cells) suggests a high feedback gain.

Although the present study demonstrates some of the advantages of fate control over renewal control, in terms of stability and disturbance rejection, it should come as no surprise that fate control also entails tradeoffs. For example, the mechanisms required to enable controlled lineage branching and high gain are likely more complex than those needed simply to regulate renewal. In addition, high-gain control forces cells of different types to appear in a sequential fashion (the higher the gain, the less overlapping the periods of production), which might not be desirable in all tissues. And when the purpose of an alternative fate is solely to control the numbers of cells that adopt a primary fate (that is, when the alternative fate is death or emigration), fate control is associated with the extra ‘overhead’ (in terms of time and materials) of producing cells that are not actually needed. Thus, the extent to which tissues are better served by renewal control or fate control is likely to depend strongly on the timing with which cells of different types are needed, the types of disturbances that are likely to arise, and the costs of implementing each type of strategy.

It should also be kept in mind that the analysis and simulations in the present study explore only some of the simplest possible implementations of renewal and fate control. A variety of additional interactions could modify or expand the options for achieving effective control; these might include feedback from non-terminal cells; alternative fates that can later be reversed (for example, quiescence; see Additional file 1: Supporting Information); a ratio of propensities to renew versus adopt a primary fate that is not held constant, but varies with feedback; positive feedback on renewal; and spatial effects (where the diffusion of feedback signals is explicitly accounted for). Exploration of these and other possibilities may be needed before detailed validation of specific control models with biological data will become practicable.

## Conclusions

Tissues achieve and maintain appropriate sizes in large part through the regulated progression of cells through lineages. Although negative feedback control of self-renewal has been shown to play an important role in this process, pure renewal control - in which differentiation along a single pathway is the only alternative - brings with it a variety of undesirable properties. Using tools from robust control theory, we show that the limitations of renewal control can be circumvented through the introduction of lineage branching, with feedback promoting alternate fate choice at the expense of both primary fate choice and self-renewal. Generic results such as these allow us to interpret common biological phenomena, such as lineage branching, sequential production of cell types, and programmed cell death, in a new light: not as mechanisms required to produce the cells of a tissue, but as hallmarks of a strategy for achieving control over cell numbers and dynamics.

## Methods

Simulations were done using the *control systems toolbox* and the ode solver *ode45* in MathWorks Matlab 8.1. The derivations of the transfer functions are shown in Additional file 1: Supporting Information.

## Box 1: Disturbance modeling and analysis

All the disturbances and parameter perturbations discussed in the main text are modeled by modifying the right-hand side of system 1 to include external disturbance Δ*(t) =* [Δ_1_*(t)*, Δ_2_*(t)*]:

4 $$ \begin{array}{l}{\dot{x}}_1=\left(2{p}_r\left({x}_2\right)-1\right)v{x}_1+{h}_1\left({x}_1,{x}_2\right){\varDelta}_1\\ {}{\dot{x}}_2=2{p}_d\left({x}_2\right)v{x}_1-d{x}_2+{h}_2\left({x}_1,{x}_2\right){\varDelta}_2\end{array} $$

We are interested in elucidating how the population of terminal cells *x*_2_ changes as a function of Δ and, in particular, how the different choices for the feedback function *p*_*r*_(∙) attenuate such disturbances. We shall explore both the static and dynamic effects of the disturbance.

We start by decomposing the disturbance into a time-independent static component $\overline{\varDelta}$ and a time-dependent component *δ(t)*, so that $\varDelta (t)=\overline{\varDelta}+\delta (t)$ (see Figure B1). The choice of $\overline{\varDelta}$ is not unique and is selected here so that the fluctuations of Δ(t) are centered around $\overline{\varDelta}$*.* If we define ${\overline{x}}_2$ to be the steady state response of the terminal cell population *x*_*2*_(*t*) only to the static component of the disturbance, then we can decompose the disturbance response as follows: ${x}_2(t)={\overline{x}}_2+{\xi}_2(t)$.

### Static disturbance response

The static disturbance response ${\overline{x}}_2$ to the constant disturbance $\overline{\Delta}$ is characterized by algebraic equations obtained by substituting $\varDelta (t)=\overline{\varDelta}$ in (4) and setting the left-hand side to zero. This relation defines a nonlinear mapping *K*_*pr*_*:*$\overline{\varDelta}\ \to {K}_{pr}\left(\overline{\varDelta}\right) = {\overline{x}}_2$. One question of interest is quantifying how the choice of *p*_*r*_(∙) affects the steady state value ${\overline{x}}_2$, as compared to ${\overline{x}}_2^{*}$, the steady state value of the undisturbed system (that is, Δ(*t*) = 0). The steady state error $\left|{\overline{x}}_2-{\overline{x}}_2^{*}\right|$ can be made small by picking *p*_*r*_(∙) with large slope *α* at steady state (see Additional file 1: Supporting Information). For instance, in Figure 2E, the error for small ${\overline{\varDelta}}_1$ is $\left|{\overline{x}}_2-{\overline{x}}_2^{*}\right|\approx \overline{\varDelta}/\left(2\alpha \right)$.

### Dynamic disturbance response

The relationship between the dynamic component of the disturbance *δ*(*t*) and the corresponding response *ξ*_*2*_(*t*) cannot be computed analytically except in very special cases. Numerical simulations (see Figures 2, 3 and 4) can be obtained for specific parameter choices, and they quickly become impractical for large numbers of parameters. However, we can study the dynamic properties of the system, often in a parameter-independent way, by examining the linearized dynamics of the system. Such dynamics approximate the nonlinear dynamics of system 4 for small disturbances δ(*t*) and present a powerful approach for studying the effects of feedback and its fundamental limitations imposed by the structural features of system 4. Under this small signal assumption, the nonlinear dynamics of system 4 are approximated by the system in Figure B1, where *W =* [*W*_*1*_ *W*_*2*_] and *L* are linear dynamical systems (see Additional file 1: Supporting Information). *W* is a weighting function that modulates how the disturbance *δ* enters the system and is independent of the feedback *p*_*r*_(∙). On the other hand, the dynamical system *S*, referred to as the ‘sensitivity function’ and defined as the feedback interconnection of the dynamical system *L* with the constant α, does depend on the *p*_*r*_. In control theory, the sensitivity function plays a fundamental role in capturing a feedback system’s stability, robustness, and disturbance-rejection properties. It also encapsulates the fundamental limitations imposed by the system’s architecture on these properties, and will therefore be the primary object of our study.

## Box 2: Performance limitations

In the frequency domain, the value *z* for which L(*z*) = 0 is called a zero of the dynamical system in Figure B1. If the real part of *z* is positive, it is called a right half-plane (RHP) zero and the system is called non-minimum phase. The behavior of non-minimum phase systems have been studied in many engineering applications [50] and more recently in autocatalytic pathways in biology, such as the glycolytic pathway [51,52]. The dynamical system *L* has a RHP zero located at *z* = *v* > 0 (see Additional file 1: Supporting Information). The existence of the RHP zero is a manifestation of the feedback and pathway structure and is independent of the choice of *p*_*r*_(*x*_*2*_). The presence of this RHP zero is at the center of performance limitations discussed in the main text and illustrated in Figure 2B-H.

As stated earlier, good rejection of disturbances is achieved by making |*S*| small at the disturbance frequency. When *L* is non-minimum phase, it is not possible to make |*S*| small across all frequencies, because *S = *(*1 + αL*)^*−1*^ must satisfy a conservation law captured by a special form of the Bode integral formula [53,54]:

5 $$ {\displaystyle \int \ln \left|S\left(j\omega \right)\right|}\frac{2v}{v^2+{\omega}^2}d\omega =0 $$

It can be shown that large α gives rise to small *|S|* at low frequencies but, as the above equation implies, *|S|* must necessarily become large at some other frequency range (Figure 2I). Hence the conservation law 5 limits the performance of the system by enforcing a tradeoff on the type of disturbances the controller can attenuate (Figure 2B-E, G). Another consequence of the existence of the RHP zero is that it places a limit on the range of gains α that lead to closed-loop system stability (stable *S*). As α approaches the stability limit, the system performance deteriorates (Figure 2E-G). This implies that even in the case of constant disturbance, high gains lead to instability and/or oscillations (Figure 2E).

There are other deleterious consequences to high-gain feedback for non-minimum phase systems. The abrupt removal of a portion of terminal cells results in dynamic changes in the population of terminal cells (known as the impulse response). For stable *S* and small proportions of terminal cells removed, the sum of the squares of deviations of the terminal cell populations from the desired steady state over time (a measure of how fast the steady state is restored, known as the *L*_*2*_-norm) is given by −0.5/(*λ*_1_ + *λ*_2_), where *λ*_1_ and *λ*_2_ are the poles of the system (that is, *S*(*λ*_*i*_) = ∞, *i* = 1, 2). The distance of the poles from the imaginary axis is a measure of how stable the system is. As the gain increases (more aggressive feedback), the system becomes less stable (the poles move closer to the imaginary axis and their sum becomes close to zero) leading to oscillatory behavior and *L*_*2*_-norm blows up.

## Additional file

Additional file 1:
**Supporting Information.** Modeling and Analysis Details. Table S1: Parameter values used in the simulations shown in Figures 2 and 4. Table S2: Parameter values used in the simulations shown in Figures 5 and 6. Figure S1: Steady state perturbations. Figure S2: Renewal control disturbance response. Figure S3: Fate control disturbance response.
