# Supplementary material for: Cell lineage branching as a strategy for proliferative control
Source: BMC Biol. 2015 Feb 19;13:13. doi: 10.1186/s12915-015-0122-8 (PMC4378012; doi:10.1186/s12915-015-0122-8)
Supplement: Additional file 1: — Supporting Information. Modeling and Analysis Details. Table S1: Parameter values used in the simulations shown in Figures 2 and 4. Table S2: Parameter values used in the simulations shown in Figures 5 and 6. Figure S1: Steady state perturbations. Figure S2: Renewal control disturbance response. Figure S3: Fate control disturbance response. [file 12915_2015_122_MOESM1_ESM.pdf]

Cell Lineage Branching as a Strategy for Proliferative Control – Supporting Information

Gentian Buzi[1,*], Arthur D. Lander[2,3], Mustafa Khammash[1,3,*]

[1]Control Theory and Systems Biology, Department of Biosystems Science and Engineering, ETH Zurich, Basel 4058, Switzerland

[2]Department of Developmental and Cell Biology, Department of Biomedical Engineering, and Center for Complex Biological Systems; University of California Irvine, Irvine, CA 92697, USA

[3]Co-senior author

*Corresponding author, gentian.buzi@bsse.ethz.ch, mustafa.khammash@bsse.ethz.ch

Disturbance modeling

All the disturbances and parameter perturbations discussed in the main text are modeled by modifying the right-hand side of (1) to include external disturbance $\Delta(t)=[\Delta_1(t), \Delta_2(t)]$

$$\begin{aligned}\dot{x}_1 &= \left(2p_r(x_2)-1\right)vx_1 + h_1(x_1,x_2)\Delta_1 \\ \dot{x}_2 &= 2p_d(x_2)vx_1 - dx_2 + h_2(x_1,x_2)\Delta_2\end{aligned} \quad \text{(S1)}$$

Here are the corresponding h_1, h_2 and Δ for some disturbance scenarios (including the ones discussed in the main text):

DS1. Cell termination at a constant rate: $h_1(x_1,x_2)=-vx_1$, $\Delta_1(t)=\bar{\Delta}_1$, $\Delta_2(t)=0$; (Figure 2E; Figure 4E),

DS2. Stochastic disturbance of stem cells only: $h_1(x_1,x_2)=vx_1$, $\Delta_1(t)=\sigma_1(t)$, $\Delta_2(t)=0$; (Figure 2G, 2H; Figure 4G, 4H; Figure S2D; Figure S3D),

DS3. Unexpected differentiation of stem cell into terminal cells: $h_1(x_1,x_2)=-vx_1$, $\Delta_1(t)=\sigma_1(t)$, $h_2(x_1,x_2)=h_1(x_1,x_2)$, $\Delta_2(t)=-\Delta_1(t)$; (Figure S2B; Figure S3B),

DS4. **C**onstant inflow of stem cells: $h_1(x_1,x_2)=1$, $\Delta_1(t)=\bar{\Delta}_1$, $\Delta_2(t)=0$; (Figure S2A; Figure S3A),

DS5. Fluctuation on the decay rate constant of differentiated cells: $\Delta_1(t)=0$, $h_2(x_1,x_2)=-x_2$, $\Delta_2(t)=\sigma_2(t)$; (Figure 2B-2D; Figure 4B-4D; Figure S2F; Figure S3F),

DS6. Abrupt removal of terminal cells: $\Delta_1(t)=0$, $h_2(x_1,x_2)=-1$, $\Delta_2(t)=\bar{\Delta}_2\delta_{Dirac}(t)$; (Figure 2F; Figure 4F),

where $\bar{\Delta}_1$, $\bar{\Delta}_2$ are constants; $\sigma_1(t)$, $\sigma_2(t)$, can be stochastic or deterministic processes; and $\delta_{Dirac}(t)$ is the Dirac delta function.

To facilitate the study of the system's response to disturbances, we start by decomposing the disturbance into a time-independent *static* component $\bar{\Delta}=[\bar{\Delta}_1, \bar{\Delta}_2]$ and a time-dependent component $\delta(t)=[\delta_1(t), \delta_2(t)]$, so that

$$\Delta(t)=\bar{\Delta}+\delta(t) \quad \text{(S2)}$$

(see Figure B1). The choice of $\bar{\Delta}$ is not unique and is selected here so that the fluctuations of $\Delta(t)$ are centered around $\bar{\Delta}$. We are interested in how the population of terminal cells x_2 changes as a function of $\Delta(t)$ and how different choices of p_r can help attenuate/reject such disturbances - i.e., minimize the perturbations of x_2 for the nominal (undisturbed, $\Delta(t)=0$) steady state value $\bar{x}_2^*$. If we define $\bar{x}_2$ to be the steady state response of the terminal cell population $x_2(t)$ only to the static component of the disturbance, then we can decompose the disturbance response as follows:

$$x_2(t) = \bar{x}_2 + \xi_2(t) \tag{S3}$$

i.e., the response of the system to the disturbance is also be decomposed into a static response $\bar{x}_2=K_{pr}(\bar{\Delta})$ and a dynamical response $\xi_2(t)=H_{pr}(t,\Delta,x(0))$ (Figure B1).

K_{pr} is a (static) nonlinear function of that characterizes the dependence of the steady state value x_2 as a function of a static disturbance (Figure S1A), $x_2 = K_{pr}(\bar{\Delta})$, and can be easily computed by solving algebraic equations:

$$\begin{aligned} 0 &= \left(2p_r(\bar{x}_2)-1\right)v\bar{x}_1 + h_1(\bar{x}_1,\bar{x}_2)\bar{\Delta}_1 \\ 0 &= 2p_d(\bar{x}_2)v\bar{x}_1 - d\bar{x}_2 + h_2(\bar{x}_1,\bar{x}_2)\bar{\Delta}_2 \end{aligned} \tag{S4}$$

where $\bar{x}_1$ is the steady state value of x_1. For small $\bar{\Delta}$, using the implicit function theorem one can show that large α (slope of p_r at steady state) minimizes $|\bar{x}_2 - \bar{x}_2^*|$ (the steady state deviation from the unperturbed state) . As an illustration, consider the scenario DS1 with $p_r(x_2) = \dfrac{1}{1.5+0.5\left(x_2/\bar{x}_2^*\right)^n}$. Then

$$K_{p_r}(\bar{\Delta}) = \left(\frac{1-3\bar{\Delta}_1}{1+\bar{\Delta}_1}\right)^{1/n}\bar{x}_2^* \text{ and } |\,\bar{x}_2-\bar{x}_2^*| = \left|1-\left(\frac{1-3\bar{\Delta}_1}{1+\bar{\Delta}_1}\right)^{1/n}\right|\bar{x}_2^* = \frac{4\bar{\Delta}_1}{n}\bar{x}_2^* + O(\bar{\Delta}_1^2) \approx \frac{\bar{\Delta}_1}{2\alpha} \text{ (Figure S1).}$$

H_{pr} is a dynamic nonlinear function of the disturbance that characterizes the behavior of the system around the steady state as a function of the disturbances. For a specific choice of parameters and disturbances one

can numerically compute H_{pr}. Such numerical computations can help us get insight into the behavior of the system (see Figures 2, 3 and 4), but do not give a complete picture and become impractical for large numbers of parameters. Analytical solutions of H_{pr} are not available (except for some very special cases). However, we can study some properties of H_{pr} by looking at the effects of small disturbances.

Note that if there are no external disturbances (i.e., $\Delta(t)=0$), then negative feedback control of p_r by x_2 produces both stability and parameter robustness of (S2). The level of terminal cells at steady state becomes determined only by the relationship between x_2 and p_r, and not by the other parameters of the system, v and d, or by initial conditions. Such “perfect” robustness is a result of integral-like control implemented by the feedback loop with integrator $\sigma = \ln x_1$ and error $e=(2\,p_r(x_2)-1)v$ (i.e., $d\sigma/dt=(2p_r(x_2)-1)v=e$). For error thus defined – a reasonable measure of deviations of terminal cell populations from the desired state – the steady state condition becomes $p_r(x_2)=1/2$, thus showing that this integral action assures perfect adaptation with respect to parameter changes of v, d, and to initial conditions.

Dynamics near steady state

Under the assumption of small disturbance $\delta(t)$, the dynamics of $\xi_2(t)$ are approximated by

$$\dot{\xi} = \begin{bmatrix} \left(2p_r(\overline{x}_2)-1\right)v & 2\dfrac{\partial p_r(\overline{x}_2)}{\partial x_2} v\overline{x}_1 \\ 2p_d(\overline{x}_2)v & 2\dfrac{\partial p_d(\overline{x}_2)}{\partial x_2} v\overline{x}_1 - d \end{bmatrix} \xi + \begin{bmatrix} \dfrac{\partial h_1}{\partial x_1}(\overline{x}_1,\overline{x}_2)\overline{\Delta}_1 & \dfrac{\partial h_1}{\partial x_2}(\overline{x}_1,\overline{x}_2)\overline{\Delta}_1 \\ \dfrac{\partial h_2}{\partial x_1}(\overline{x}_1,\overline{x}_2)\overline{\Delta}_2 & \dfrac{\partial h_2}{\partial x_1}(\overline{x}_1,\overline{x}_2)\overline{\Delta}_2 \end{bmatrix} \xi + \begin{bmatrix} h_1(\overline{x}_1,\overline{x}_2)\delta_1 \\ h_2(\overline{x}_1,\overline{x}_2)\delta_2 \end{bmatrix}$$

subject to (S4), which can be rewritten as:

$$\dot{\xi} = \overbrace{\begin{bmatrix} 0 & 2\frac{\partial p_r(\bar{x}_2)}{\partial x_2} v\bar{x}_1 \\ d\frac{1}{\bar{x}_1}\bar{x}_2 & 2\frac{\partial p_d(\bar{x}_2)}{\partial x_2} v\bar{x}_1 - d \end{bmatrix}}^{A_\alpha}\xi + \overbrace{\begin{bmatrix} \left(\frac{\partial h_1}{\partial x_1}(\bar{x}_1,\bar{x}_2) - \frac{1}{\bar{x}_1}h_1(\bar{x}_1,\bar{x}_2)\right)\bar{\Delta}_1 & \frac{\partial h_1}{\partial x_2}(\bar{x}_1,\bar{x}_2)\bar{\Delta}_1 \\ \left(\frac{\partial h_2}{\partial x_1}(\bar{x}_1,\bar{x}_2) - \frac{1}{\bar{x}_1}h_2(\bar{x}_1,\bar{x}_2)\right)\bar{\Delta}_2 & \frac{\partial h_2}{\partial x_1}(\bar{x}_1,\bar{x}_2)\bar{\Delta}_2 \end{bmatrix}}^{A_\Delta}\xi$$
$$+\overbrace{\begin{bmatrix} h_1(\bar{x}_1,\bar{x}_2) & 0 \\ 0 & h_2(\bar{x}_1,\bar{x}_2) \end{bmatrix}}^{B_\delta}\begin{bmatrix}\delta_1 \\ \delta_2\end{bmatrix}.$$

For disturbance DS4, $A_\Delta = \begin{bmatrix} -\frac{1}{\bar{x}_1}\bar{\Delta}_1 & 0 \\ 0 & 0 \end{bmatrix}$. For all the other disturbances A_Δ=0 and $\bar{x}_2 d/\bar{x}_1 = v$. Next we consider the case when A_Δ=0 (i.e., all disturbances except for DS4). The analysis of DS4 yields the same conclusions (the functions are a bit messier).

The renewal control model ($p_d(x_2) = 1 - p_r(x_2)$) is given by

$$\dot{\xi} = \underbrace{\overbrace{\begin{bmatrix} 0 & 0 \\ d\frac{1}{\bar{x}_1}\bar{x}_2 & -d \end{bmatrix}}^{A_0}}_{\text{open loop dynamics}}\xi \underbrace{-\alpha\overbrace{\begin{bmatrix} 2v\bar{x}_1 \\ -2v\bar{x}_1 \end{bmatrix}}^{B_\alpha}\xi}_{\text{control}} + \underbrace{\overbrace{\begin{bmatrix} h_1(\bar{x}_1,\bar{x}_2) & 0 \\ 0 & h_2(\bar{x}_1,\bar{x}_2) \end{bmatrix}}^{B_\delta}\delta}_{\text{disturbance}} \qquad \text{(S5)}$$

and the fate control model ($p_d(x_2) = \kappa p_r(x_2)$) by

$$\dot{\xi} = \underbrace{\overbrace{\begin{bmatrix} 0 & 0 \\ d\frac{1}{\bar{x}_1}\bar{x}_2 & -d \end{bmatrix}}^{A_0}}_{\text{open loop dynamics}}\xi \underbrace{-\alpha\overbrace{\begin{bmatrix} 2v\bar{x}_1 \\ \kappa 2v\bar{x}_1 \end{bmatrix}}^{B_\alpha}\xi_2}_{\text{control}} + \underbrace{\overbrace{\begin{bmatrix} h_1(\bar{x}_1,\bar{x}_2) & 0 \\ 0 & h_2(\bar{x}_1,\bar{x}_2) \end{bmatrix}}^{B_\delta}\delta}_{\text{disturbance}}, \qquad \text{(S6)}$$

where A_0 describes the open loop dynamics (no feedback), and $\alpha := -\frac{\partial p_r(\bar{x}_2)}{\partial x_2}$ is the slope of p_r at steady state.

The dynamical systems for both fate and renewal control models can be more instructive in the Laplace (frequency) domain, where it also reveals some non-trivial properties of the system. Indeed, the effect of the disturbances δ_1, δ_2 on ξ_2 (the perturbations of population of terminal cells from its nominal value) for renewal control is given by

$$\xi_2(s) = G_1(s)\delta_1(s) + G_2(s)\delta_2(s) \tag{S7}$$

where s is the frequency (Laplace) independent variable and

$$\begin{aligned} G_1(s) &= C\left(sI - \left(A_0 - \alpha B_\alpha C\right)\right)^{-1} B_\delta^{(1)} \\ G_2(s) &= C\left(sI - \left(A_0 - \alpha B_\alpha C\right)\right)^{-1} B_\delta^{(2)} \end{aligned}$$

where $C = [\ 0\ 1\ ]$, $B_\delta^{(i)}$ is the i-th column of B_δ, and I is the identity matrix.

For $s \neq 0$, $s \neq -d$, we rewrite $G_1 = W_1 S$, and $G_2 = W_2 S$ where

$$\begin{aligned} \mathrm{S}(s) &= \frac{1}{1+\alpha L(s)} \\ \mathrm{L}(s) &= C\left(sI - A_0\right)^{-1} B_\alpha \end{aligned}$$

and

$$\begin{aligned} W_1(s) &= \frac{v}{s(s+d)} h_1(\overline{x}_1, \overline{x}_2) \\ W_2(s) &= \frac{1}{s+d} h_2(\overline{x}_1, \overline{x}_2) \end{aligned}$$

(Figure B1). S is called the sensitivity function of the closed loop of system (1), and L is called the open loop transfer function (or the open loop plant).

Consider the case when the disturbance is a sinusoid $\delta_1(t) = \sin\omega_1 t$. Then $||\xi_2||_\infty :=\sup_t \xi_2(t)$, the maximum perturbation of the terminal cells from the nominal value x_2, is given by $|G_1(j\omega_1)|$ where $j = \sqrt{}$, i.e., the

magnitude of G_1 at the frequency ω_1. Similarly if $\delta_2(t) = \sin\omega_2 t$, $||\xi_2||_\infty = |G_2(j\omega_2)|$. In general, any disturbance can be expressed as a sum of sinusoidals and the sinusoidal frequencies ω compose what is called the disturbance spectrum. So for a general disturbance, the reduction of the perturbations $||\xi_2||_\infty$ is dependent on the magnitude of G_1 and G_2 across the disturbance spectrum. Let $\|G\|_\infty := \max_{j\omega}|G(j\omega)|$. Then $\|G_1\|_\infty$ and $\|G_2\|_\infty$ are the maximum perturbation from the nominal value for any frequency of δ_1 and δ_2 respectively.

W_1 and W_2 are independent of p_r, and therefore the question of which choice of p_r best rejects/attenuates a disturbance δ is equivalent to which choice of p_r best reduces the magnitude of S across the disturbance spectrum. We show that for the branched topology, there are constrains that limit how small S can be made.

Renewal control model

In this case the specific transfer functions are given by

$$
\begin{aligned}
G_1(s) &= \frac{v}{s^2 + (d - 2\alpha\bar{x}_1 v)s + 2\alpha v d\bar{x}_2} h_1(\bar{x}_1, \bar{x}_2) \\
G_2(s) &= \frac{s}{s^2 + (d - 2\alpha\bar{x}_1 v)s + 2\alpha v d\bar{x}_2} h_2(\bar{x}_1, \bar{x}_2) \\
S(s) &= \frac{s(s+d)}{s^2 + (d - 2\alpha\bar{x}_1 v)s + 2\alpha v d\bar{x}_2} \\
L(s) &= 2\bar{x}_1 v \frac{v - s}{s(s+d)}
\end{aligned}
$$

and

$$
\begin{aligned}
W_1(s) &= \frac{v}{s(s+d)} h_1(\bar{x}_1, \bar{x}_2) \\
W_2(s) &= \frac{1}{s+d} h_2(\bar{x}_1, \bar{x}_2).
\end{aligned}
$$

If $L(z) = 0$ then z is called the zeros of the system and if $S(\lambda) = \infty$, λ is called a pole of the closed loop system. In this case $z = v > 0$ is a zero of the system, and the system is called non-minimum phase (since $z > 0$ is a right half-plane (RHP) zero). The steady state error to a small constant perturbation disturbance δ_1 is given by $|\xi_2/\delta_1|=G_1(0)=h_1(\bar{x}_1, \bar{x}_2)/(2\alpha d\bar{x}_2)$, i.e., it is inversely proportional to the gain α. Therefore, more aggressive controllers result in smaller errors for disturbances of this type. In general we want G_1 and G_2 to be small across all frequencies ω (not just $\omega = 0$). However the existence of the RHP zero $z = v$, imposes some hard constraints on how small G_1 and G_2 can be made. First, using maximum modulus theorem can be shown that

$$\|G_1\|_\infty = \max_{Re(s\geq 0)} |G_1(s)| \geq W_1(z) = \frac{1}{d+v} h_1(\bar{x}_1, \bar{x}_2),$$
$$\|G_2\|_\infty = \max_{Re(s\geq 0)} |G_2(s)| \geq W_2(z) = \frac{1}{d+v} h_2(\bar{x}_1, \bar{x}_2),$$

and therefore, there is a limit to how small the peaks of G_1 and G_2 can be made, independent of the choice of controller.

Furthermore, S must satisfy a special form of Bode's integral formula

$$\int \ln|S(j\omega)| \frac{2v}{v^2+\omega^2} d\omega = 0 \quad \text{(S8)}$$

Since the weights W_1 and W_2 are independent of the controller, then (S8) is a general constraint on G_1 and G_2 ($\ln|G_i(j\omega)| = \ln|W_i(j\omega)| + \ln|S(j\omega)|$, $i = 1, 2$). $|S(j\omega)| < 1$ implies an attenuation of noise at frequency ω, while $|S(j\omega)| > 1$ implies and amplification of noise at frequency ω. Equation (S8) is a type of conservation law, stating that the net disturbance attenuation and amplification must be balanced. The weight $2v$ in (S8) is a low pass filter, which makes the disturbance attenuation at low frequencies more costly (i.e., higher amplification at other frequencies).

Fate control model

In this case the specific transfer functions are given by

$$
\begin{aligned}
G_1(s) &= \frac{\kappa v}{s^2 + (d + 2\alpha \bar{x}_1 v\kappa)s + 2\alpha v d\bar{x}_2} h_1(\bar{x}_1, \bar{x}_2) \\
G_2(s) &= \frac{s}{s^2 + (d + 2\alpha \bar{x}_1 v\kappa)s + 2\alpha v d\bar{x}_2} h_2(\bar{x}_1, \bar{x}_2) \\
S(s) &= \frac{s(s+d)}{s^2 + (d + 2\alpha \bar{x}_1 v\kappa)s + 2\alpha v d\bar{x}_2} \\
L(s) &= 2\bar{x}_1 v\kappa \frac{v+s}{s(s+d)}
\end{aligned}
$$

and

$$
\begin{aligned}
W_1(s) &= \frac{\kappa v}{s(s+d)} h_1(\bar{x}_1, \bar{x}_2) \\
W_2(s) &= \frac{1}{s+d} h_2(\bar{x}_1, \bar{x}_2).
\end{aligned}
$$

Unlike renewal control, this system does not have a RHP zero (the zero is at $z = -v$). The Bode integral formula does not hold (since L is relative degree 1) and there are no restrictions on how strong the stabilizing feedback can be used (all positive gains are stabilizing).

Regulated cell cycle rate for unbranched lineages

Consider renewal control, where there is some additional regulation of the rate at which cells divide, i.e., v is a function of x_2. The dynamics given by

$$
\begin{aligned}
\dot{x}_1 &= \left(2p_r(x_2) - 1\right) v(x_2) x_1 \\
\dot{x}_2 &= 2p_d(x_2) v(x_2) x_1 - dx_2
\end{aligned}
\tag{S9}
$$

subject to

$$p_d(x_2) = 1 - p_r(x_2).$$

Let $v{:} = v(\bar{x}_2)$ and $\beta = -\partial v(\bar{x}_2)\partial\bar{x}_2$. For a given β, the dynamics of (S9) near the steady state are given by

$$\dot{\xi} = \overbrace{\begin{bmatrix} 0 & 2\dfrac{\partial p_r(\overline{x}_2)}{\partial x_2}\overline{v}\overline{x}_1 \\ d\dfrac{1}{\overline{x}_1}\overline{x}_2 & 2\alpha\overline{v}\overline{x}_1 - \beta\overline{x}_1 - d \end{bmatrix}}^{A_\alpha}\xi.$$

Consider small disturbances δ_1, δ_2

$$\dot{\xi} = \underbrace{\overbrace{\begin{bmatrix} 0 & 0 \\ d\dfrac{1}{\overline{x}_1}\overline{x}_2 & -d - \beta\overline{x}_1 \end{bmatrix}}^{A_0}\xi}_{\text{open loop dynamics}} \underbrace{-\alpha\overbrace{\begin{bmatrix} 2\overline{v}\overline{x}_1 \\ -2\overline{v}\overline{x}_1 \end{bmatrix}}^{B_\alpha}\xi_2}_{\text{control}} + \underbrace{\begin{bmatrix} \delta_1 \\ \delta_2 \end{bmatrix}}_{\text{disturbance}}.$$

The response to these disturbances is given by the transfer functions $G_1 = W_1S$ and $G_2 = W_2S$ (for δ_1 and δ_2 respectively), where

$$S(s) = \frac{s(s + d + \beta\overline{x}_1)}{s^2 + (d + \beta\overline{x}_1 - 2\alpha\overline{x}_1\overline{v})s + 2\alpha\overline{v}d\overline{x}_2},$$

and

$$W_1(s) = \frac{v}{s(s + d + \beta\overline{x}_1)},$$
$$W_2(s) = \frac{1}{s + d + \beta\overline{x}_1},$$

and $S = \dfrac{1}{1+\alpha L}$, $L(s) = 2\overline{x}_1\overline{v}\dfrac{\overline{v} - s}{s(s + d + \beta\overline{x}_1)}$. L still has a zero in the RHP at $s = v$ (rate of cell cycle at steady state). The extra regulation β is beneficial if $\beta > 0$ (i.e., negative regulation of cell cycle rate), since

it allows for larger stable gains α (i.e., stable gains are $0 < \alpha < b + \beta x_1$), but it does not remove the fundamental limitations imposed by the RHP zero.

Trans-differentiation or delayed differentiation as a “temporary” alternate fate

Here we consider a branched lineage in which the alternative differentiated cell fate x_3, at some later time, reconnects back to the original differentiated fate, either through trans-differentiation or delayed differentiation so that ultimately there is only one “terminally" differentiated cell type (x2). Might this scheme reap the benefits of fate control without incurring the extra “cost” of producing a potentially unnecessary alternative cell type?

The ODE model for such a scheme would be given by

$$\begin{aligned} \dot{x}_1 &= \left(2p_r(x_2)-1\right)v(x_2)x_1 \\ \dot{x}_2 &= 2p_d(x_2)v(x_2)x_1 - dx_2 + ux_3 \\ \dot{x}_3 &= 2p_a(x_2)v(x_2)x_1 - ux_3 \end{aligned} \tag{S10}$$

where u is the rate at which x_3 differentiate (or trans-differentiate) into x_2 (we can add an additional death rate term into the third equation, but it doesn’t change the substance of the argument that follows). If we apply the fate control scheme described above, with $p_d=\kappa p_r$ and negative feedback on p_r, we find that as u gets very small, these equations approach the basic fate control model, whereas as u gets very large, the third equation becomes increasingly irrelevant, and the system approaches the renewal control model.

The sensitivity function is given by

$$S(s) = \frac{s^3+(d+u)s^2+dus}{s^3+(d+u+2\alpha\bar{x}_1 v\kappa)s^2+(du+2\alpha v\bar{x}_1(v\kappa-u))s+2\alpha u v^2\bar{x}_1}.$$

For $u>\kappa v$ the system has two RHP zeros and the limitations of non-minimum phase systems apply. On the other hand, for $u<\kappa v$, the zeros move to the LHP and the system is minimum phase. However, removal of the RHP zeros is necessary, but not sufficient, for overcoming the performance limitations of renewal control. Specifically for u close to κv, large gains destabilize the system (i.e., S has unstable poles in the RHP). It is only for u much smaller than v and d that high gains become stabilizing and the performance

resembles that of a true fate-controlled system with non-reconnecting branches. In other words, if the rate of trans-differentiation (or delayed differentiation) can be made sufficiently slow compared to the rate of the cell cycle (i.e. if cells hang around in the not-yet-fully differentiated state for many cell cycles), then robustness to disturbances can be achieved as if the situation was one of pure fate control. However, there is a large price for this: Slow u implies that the population of transient cells x_3 at steady state is large (since $\bar{x}_3 \sim 1/u$). Thus, only a fraction of the tissue can be composed of terminally differentiated cells at steady state (the larger the fraction, the poorer the performance). If the tissue is of the "final-state" type, then of course the x_3 cells do all eventually turn into x_2 cells, but the requirement for slow u means that they can only do so very slowly (without the final tissue size robustness of fate control). So, either way, the use of delayed or trans-differentiation to avoid permanent lineage branching brings with it either a large overhead of unnecessary cells, or a dramatic slowing of tissue generation and final tissue size sensitivity.

Layered pathways

The dynamics given by

$$\begin{aligned}
\dot{x}_1 &= \left(2p_r^{(1)}(x_2)-1\right)v_1x_1 + h^{(1)}(x_1)\Delta^{(1)} \\
\dot{x}_2 &= 2p_d^{(1)}(x_2)v_1x_1 - d_1x_2 \\
\dot{x}_3 &= 2p_a^{(1)}(x_2)v_1x_1 + \left(2p_r^{(2)}(x_4)-1\right)v_2x_3 + h^{(2)}(x_3)\Delta^{(2)} \\
\dot{x}_4 &= 2p_d^{(2)}(x_4)v_2x_3 - d_2x_4 \\
\dot{x}_5 &= 2p_a^{(2)}(x_4)v_2x_3 + \left(2p_r^{(3)}(x_6)-1\right)v_3x_5 + h^{(3)}(x_5)\Delta^{(3)} \\
\dot{x}_6 &= 2p_d^{(3)}(x_6)v_3x_5 - d_3x_6,
\end{aligned} \qquad \text{(S11)}$$

subject to

$$p_a^{(i)}(x_{2i}) = 1 - p_r^{(i)}(x_{2i}) - p_d^{(i)}(x_{2i}), \;\; i = 1, 2, 3.$$

Supporting Figure Legends

Figure S1. *Steady state perturbations.* (**A**) The new steady state population $\bar{x}_2$ as a function (K_{pr}) of the static disturbance $\bar{\Delta}$. For aggressive feedback (large α) $\bar{x}_2$ is close to the unperturbed steady state $\bar{x}_2^*$.

Shown is scenario DS1 ($h_1(x)=-vx_1$, $\Delta_1(t)=\bar{\Delta}_1$, $\Delta_2(t)=0$) with $p_r(x_2)=\dfrac{1}{1.5+0.5(x_2/100)^n}$, v=1 , d=0.1 and n=1,2,4,10 . (**B**) For small $\bar{\Delta}$, the perturbation from the unperturbed steady state is inversely proportional to the slope of p_r . Shown is a geometrical representation of this relationship for DS1 (constant removal of stem cells), resulting in $|\bar{x}_2 - \bar{x}_2^*| \approx \bar{\Delta}_1/(2\alpha)$.

Figure S2. *Renewal control disturbance response.* (**A**) Scenario DS4. Aggressive feedback improves the steady state error $|\bar{x}_2 - \bar{x}_2^*|$ but also induce oscillatory behavior. (**B**) Scenario DS3. Low levels of feedback can improve both the static error and dynamic variability of the response. For more aggressive feedback smaller the steady state error is countered by increased variability of the response. Shown in the left panel is the response for a single sample path realization of the stochastic process. In the right panel is shown the mean and the standard deviation for 5 different realizations. (**C**) The transmission function W_1S for disturbances of type δ_1 shows a tradeoff on the ability of the controller to reject low frequency components of δ_1 by increasing the gain α , at the expense of amplifying frequencies components at a different range. (**D**) Scenario DS2 (δ_1 type disturbance). Reduction in variance is seen for low gains, but as gains increase so is the variability of the response. Shown in the left panel is the response for a single sample path realization of the stochastic process. The mean and the standard deviation for 5 different realizations are shown in the right panel. (**E**) The transmission function W_2S for disturbances of type δ_2 shows a tradeoff on the ability of the controller to reject low frequency components of δ_2 by increasing the gain α , at the

expense of amplifying frequencies components at a different range. (**F**) Scenario DS5 (δ_2 type disturbance). Reduction in variance is seen for low gains, but as gains increase so is the variability of the response. Shown in the left panel is the response for a single sample path realization of the stochastic process. The mean and the standard deviation for 5 different realizations are shown in the right panel.

Figure S3. *Fate control disturbance response.* (**A**) Scenario DS4. Aggressive feedback improves the steady state error $|\bar{x}_2 - \bar{x}_2^*|$ without inducing oscillations. (**B**) Scenario DS3. Aggressive feedback results in both smaller steady state error and decreased variability of the response. Shown in the left panel is the response for a single sample path realization of the stochastic process. The mean and the standard deviation for 5 different realizations are shown in the right panel. (**C**) The transmission function W_1S for disturbances of type δ_1 shows that for high gains α can reduce all disturbance frequencies. (**D**) Scenario DS2 (δ_1 type disturbance). High gains decrease the variability of the response. Shown in the left panel is the response for a single sample path realization of the stochastic process. In the right panel is shown the mean and the standard deviation for 5 different realizations. (**E**) The transmission function W_2S for disturbances of type δ_2 shows that for high gains α can reduce all disturbance frequencies. (**F**) Scenario DS5 (δ_2 type disturbance). High gains decrease the variability of the response. Shown in the left panel is the response for a single sample path realization of the stochastic process. The mean and the standard deviation for 5 different realizations are shown in the right panel.

Supporting Tables

Table S1. *Parameter values used in the simulations shown in Figures 2 and 4.*

Figure	Panel	Description	*v*	*d*	h_1	Δ_1	h_2	Δ_2
Figure 2	b	DS5	1	0.1	0	0	$-x_2$	d-0.1d $\eta_2(t)$
	c	Oscillations in TD death	1	0.1	0	0	$-x_2$	0.3d sin(0.02πt)
	d	Oscillations in TD death	1	0.1	0	0	$-x_2$	0.3d sin(0.08πt)
	e	DS1	1	0.1	0.15x_1	1	0	0
	f	DS6	1	0.1	-	-	-	-
	g, h	DS2	1	0.1	x_1	0.01$\eta_1(t)$-1	0	0
	i	|*S*|	1	0.1	-	-	-	-
Figure 4	b	DS5	2	0.1	0	0	$-x_2$	d-0.1d $\eta_2(t)$
	c	Oscillations in TD death	1	0.05	0	0	$-x_2$	0.3d sin(0.02πt)
	d	Oscillations in TD death	1	0.05	0	0	$-x_2$	0.3d sin(0.08πt)
	e	DS1	1	0.05	0.15x_1	1	0	0
	f	DS6	1	0.05	-	-	-	-
	g,h	DS2	1	0.05	x_1	0.01$\eta_1(t)$-1	0	0
	i	|*S*|	1	0.05	-	-	-	-

The dynamics are given by Eq. (S1) with $p_r(x_2) = \dfrac{1}{1.5 + 0.5\left(x_2 / \overline{x}_2^*\right)^n}$, $\overline{x}_2^* = 100$. For the branched lineage (Figure 4) $p_d(x_2)$=0.5 $p_r(x_2)$. The disturbances enter the system at t=0. At t=0 the system is at steady state $x_1(0)$=10, $x_2(0)$=100, except for Figure 2G and 4G where $x_1(0)$=10, $x_2(0)$=50. $\eta_1(t)$ is a birth-death process with birth rate 2.5 and death rate 0.025. η_2(t) is a birth-death process with birth rate 2.5 and death rate 0.25. DS1, DS2, DS5 and DS6 are disturbance types described in the Supporting Information.

Table S2. *Parameter values used in the simulations shown in Figures 5 and 6.*

	**Figure 5**		**Figure 6**	
**Panel**	**A,C**	**B,D**	**E**	**F**
**Description**	*renewal control*	*fate control*	*renewal control*	*fate control*
$p_r^{(1)}$	$\frac{0.9}{1.4+0.4(x_2/100)^2}$	$\frac{1}{1.8+0.2(x_2/100)^{100}}$	$\frac{0.9}{1.1+0.9(x_2/7663.1)^{100}}$	$\frac{1}{1.5+0.5(x_2/57294)^{100}}$
$p_d^{(1)}$	0.9- $p_r^{(1)}$	0.8 $p_r^{(1)}$	0.9- $p_r^{(1)}$	0.5 $p_r^{(1)}$
$p_r^{(2)}$	$\frac{0.85}{1.35+0.35(x_4/50.41)^2}$	$\frac{1}{1.8+0.2(x_4/74.07)^{100}}$	$\frac{0.9}{1.1+0.9(x_4/2300.1)^{100}}$	$\frac{1}{1.5+0.5(x_4/42704)^{100}}$
$p_d^{(2)}$	0.85- $p_r^{(2)}$	0.8 $p_r^{(2)}$	0.9- $p_r^{(2)}$	0.5 $p_r^{(2)}$
$p_r^{(3)}$	$\frac{1}{1.5+0.5(x_6/29.07)^2}$	$\frac{1}{1.8+0.2(x_6/57.7)^{100}}$	$\frac{0.9}{1.1+0.9(x_6/1533.1)^{100}}$	$\frac{1}{1.5+0.5(x_6/28201)^{100}}$
$p_d^{(3)}$	1- $p_r^{(3)}$	0.8 $p_r^{(3)}$	0.9- $p_r^{(3)}$	0.5 $p_r^{(3)}$
(v_1,v_2,v_3)	(1,1,1)	(1,1,1)	(1,1,1)	(1,1,1)
(d_1,d_2,d_3)	(0.06,0.09,0.121)	(0.06,0.064,0.0107)	(0,0,0)	(0,0,0)
$h_1^{(1)}(x_1)\Delta^{(1)}$	$(0.01\eta^{(1)}(t)\text{-}1)\ x_1$	$(0.01\eta^{(1)}(t)\text{-}1)\ x_1$	$-\rho x_1$ (dotted plot)	$-\rho x_1$ (dotted plot)
$h_1^{(2)}(x_3)\Delta^{(2)}$	$(0.01\eta^{(2)}(t)\text{-}1)\ x_3$	$(0.01\eta^{(2)}(t)\text{-}1)\ x_3$	$-\rho x_3$ (dotted plot)	$-\rho x_3$ (dotted plot)
$h_1^{(3)}(x_5)\Delta^{(3)}$	$(0.01\eta^{(3)}(t)\text{-}1)\ x_5$	$(0.01\eta^{(3)}(t)\text{-}1)\ x_5$	$-\rho x_5$ (dotted plot)	$-\rho x_5$ (dotted plot)

The dynamics are given by Eq. (S10). The disturbances enter the system at t=0. For simulations in Figure 5, at t=0 the system is at the steady state x_1=7.5, x_2=100, x_3=7.5, x_4=75, x_5=5, x_6=60 and $\eta^{(i)}$ (t) , i=1,2,3 are birth-death processes with birth rate 2.5 and death rate 0.025 . For simulations in Figure 6E-6F, the initial population of stem cells x_1 is 10 and there are no other types of cells. The desired final concentrations are 60000, 45000, and 30000 for x_2, x_4, and x_6 respectively.

Table S3. *Parameter values used in the simulations shown in Figures S2 and S3.*

Figure	Panel	Description	v	d	h_1	Δ_1	h_2	Δ_2
Figure S2	a	DS4	1	0.1	1	1	0	0
	b	DS3	1	0.1	x_1	$0.015\eta_2(t)$	x_1	$-\Delta_1$
	c	$\|W_1S\|$	1	0.1	-	-	-	-
	d	DS2	1	0.1	x_1	$0.01\eta_1(t)$-1	0	0
	e	$\|W_2S\|$	1	0.1	-	-	-	-
	f	DS5	1	0.1	0	0	$-x_2$	d-0.1d $\eta_2(t)$
Figure S3	a	DS4	1	0.05	1	1	0	0
	b	DS3	1	0.05	x_1	$0.015\eta_2(t)$	x_1	$-\Delta_1$
	c	$\|W_1S\|$	1	0.05	-	-	-	-
	d	DS2	1	0.05	x_1	$0.01\eta_1(t)$-1	0	0
	e	$\|W_2S\|$	1	0.05	-	-	-	-
	f	DS5	2	0.1	0	0	$-x_2$	d-0.1d $\eta_2(t)$

The dynamics are given by Eq. (S1) with $p_r(x_2) = \dfrac{1}{1.5 + 0.5\left(x_2 / \overline{x}_2^*\right)^n}$, $\overline{x}_2^* = 100$. For the branched lineage (Figure 4) $p_d(x_2)$=0.5 $p_r(x_2)$. The disturbances enter the system at t=0 . At t=0 the system is at steady state $x_1(0)$=10, $x_2(0)$=100. $\eta_1(t)$ is a birth-death process with birth rate 2.5 and death rate 0.025. η_2(t) is a birth-death process with birth rate 2.5 and death rate 0.25. DS1, DS2, DS5 and DS6 are disturbance types described in the Supporting Information.

Supporting Figures

Figure S1

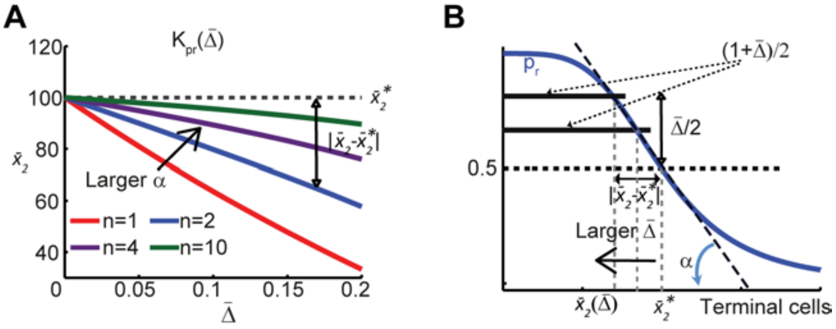

Figure S2

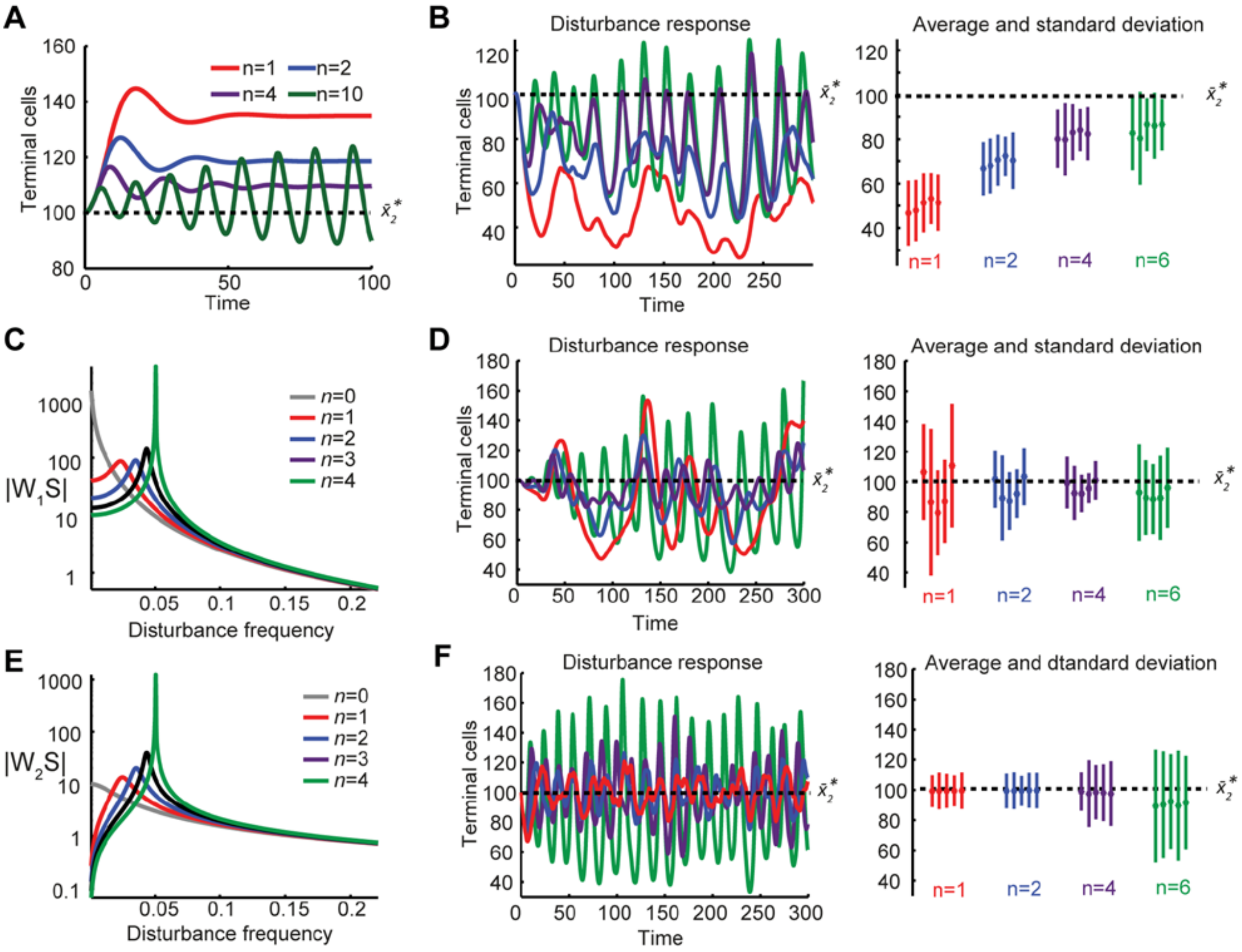

A
n=1
n=2
n=4
n=10
Terminal cells
Time
B
Disturbance response
Terminal cells
Time
Average and standard deviation
n=1
n=2
n=4
n=6
C
n=0
n=1
n=2
n=3
n=4
$|W_1S|$
Disturbance frequency
D
Disturbance response
Terminal cells
Time
Average and standard deviation
n=1
n=2
n=4
n=6
E
n=0
n=1
n=2
n=3
n=4
$|W_2S|$
Disturbance frequency
F
Disturbance response
Terminal cells
Time
Average and dtandard deviation
n=1
n=2
n=4
n=6

Figure S3

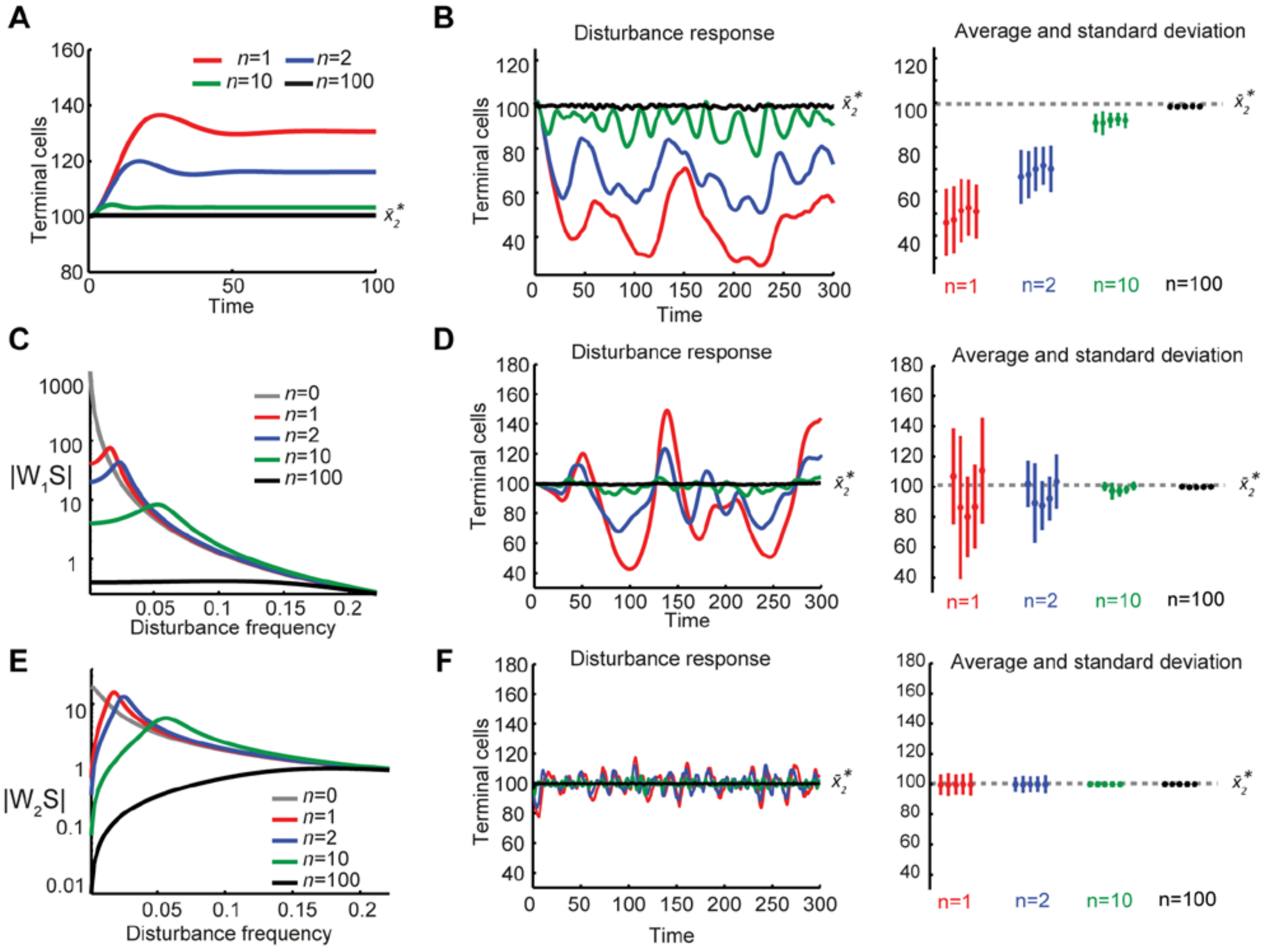
A
B
C
D
E
F
Terminal cells
Time
n=1
n=2
n=10
n=100
n=0
Disturbance response
Average and standard deviation
|W1S|
|W2S|
Disturbance frequency
